# Phage-based peptides for pancreatic cancer diagnosis and treatment: alternative approach

**DOI:** 10.3389/fmicb.2023.1231503

**Published:** 2023-08-02

**Authors:** Yang Li, Kai-di Yang, Hao-yu Duan, Ya-nan Du, Jun-feng Ye

**Affiliations:** ^1^General Surgery Center, First Hospital of Jilin University, Changchun, China; ^2^School of Nursing, Jilin University, Changchun, China

**Keywords:** pancreatic cancer, phage display, peptide therapy, cancer targeting, early detection

## Abstract

Pancreatic cancer is a devastating disease with a high mortality rate and a lack of effective therapies. The challenges associated with early detection and the highly aggressive nature of pancreatic cancer have limited treatment options, underscoring the urgent need for better disease-modifying therapies. Peptide-based biotherapeutics have become an attractive area of research due to their favorable properties such as high selectivity and affinity, chemical modifiability, good tissue permeability, and easy metabolism and excretion. Phage display, a powerful technique for identifying peptides with high affinity and specificity for their target molecules, has emerged as a key tool in the discovery of peptide-based drugs. Phage display technology involves the use of bacteriophages to express peptide libraries, which are then screened against a target of interest to identify peptides with desired properties. This approach has shown great promise in cancer diagnosis and treatment, with potential applications in targeting cancer cells and developing new therapies. In this comprehensive review, we provide an overview of the basic biology of phage vectors, the principles of phage library construction, and various methods for binding affinity assessment. We then describe the applications of phage display in pancreatic cancer therapy, targeted drug delivery, and early detection. Despite its promising potential, there are still challenges to be addressed, such as optimizing the selection process and improving the pharmacokinetic properties of phage-based drugs. Nevertheless, phage display represents a promising approach for the development of novel targeted therapies in pancreatic cancer and other tumors.

## Graphical abstract

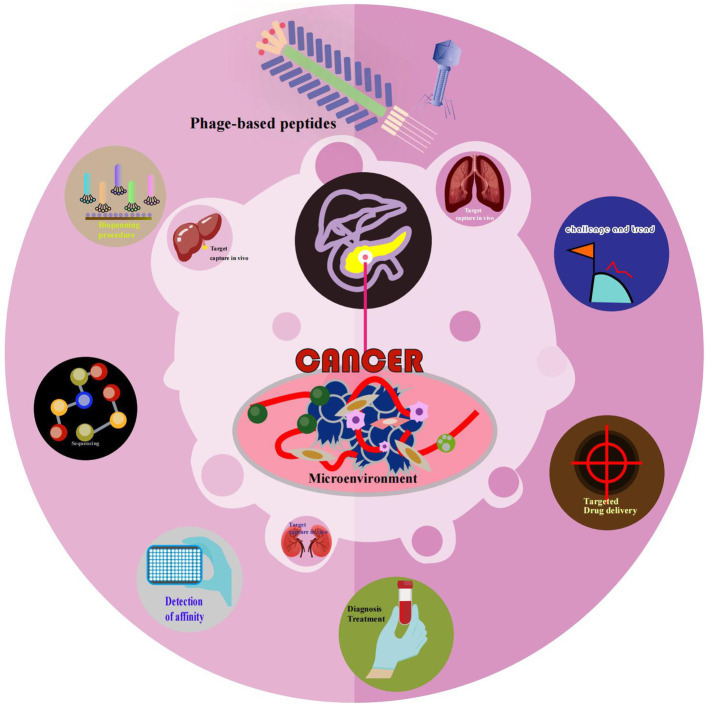

## Introduction

**Figure 1:**
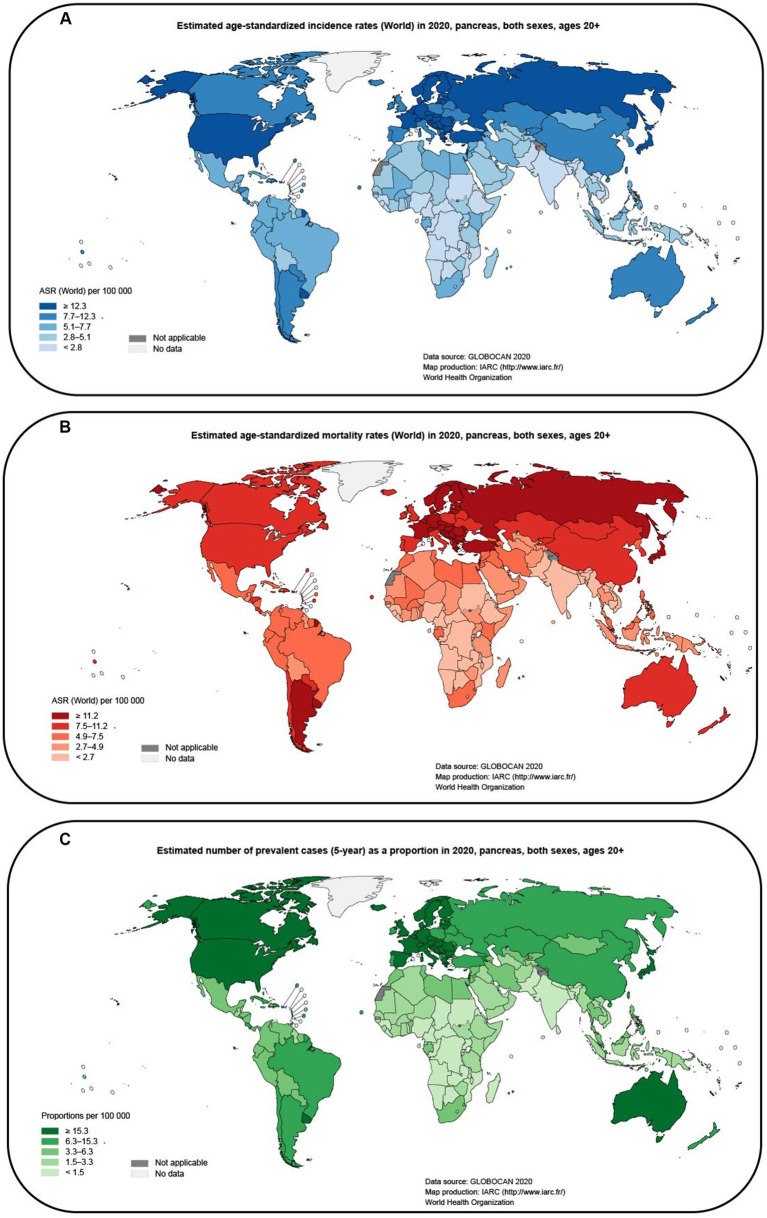
Map of global pancreatic cancer incidence and mortality estimated from GLOBOCAN (2020; data from gco.iarc.fr). **(A)** Estimated age-standardized incidence rates (World) in 2020, pancreas, both sexes, ages 20+. **(B)** Estimated age-standardized mortality rates (World) in 2020, pancreas, both sexes, ages 20+. **(C)** Estimated number of prevalent cases (5-year) as a proportion in 2020, pancreas, both sexes, ages 20 + .

Pancreatic cancer is a highly aggressive and lethal malignancy with a poor prognosis compared to other cancers (Li et al., 2019). It is often detected at an advanced stage, leading to limited treatment options and a low overall survival rate (Chen H. et al., 2021). According to GLOBOCAN (2020), the incidence and mortality rates of pancreatic cancer remain high, affecting both men and women globally. These statistics highlight the urgent need for improved methods of detection and more effective treatments for pancreatic cancer. It is estimated that pancreatic cancer is responsible for approximately 460,000 deaths per year, and there are nearly 490,000 new cases annually (Sung et al., 2021). The poor prognosis of pancreatic cancer has resulted in it being one of the leading causes of cancer-related deaths (Klein, 2021; Figures 1A–C). Furthermore, the economic burden associated with pancreatic cancer has also increased over the past few decades. This highlights the urgent need to find better treatment options for patients with pancreatic cancer. Despite the development of various treatment options, the overall prognosis of patients with pancreatic cancer remains poor. Radical surgery is currently the only curative option (Casolino et al., 2021), but most patients are diagnosed at an advanced stage when surgery is not feasible (Tonini and Zanni, 2021). Single-agent immunotherapies such as immune checkpoint inhibitors, CAR-T therapy, and specific antigen vaccines have achieved remarkable success in other cancers but are not as effective in pancreatic cancer (Morrison et al., 2018). Immunotherapies have shown limited efficacy, and combination therapy is associated with increased toxicity and cost (Schizas et al., 2020). The targeted drug erlotinib in combination with gemcitabine has only demonstrated a modest improvement in overall survival (Ettrich and Seufferlein, 2021). The stroma-targeting drug PEGPH20 combined with NAB-paclitaxel and gemcitabine has shown promise in improving progression-free survival, particularly in patients with high tumor hyaluronan levels (Neoptolemos et al., 2018), but the combination with mFOLFIRINOX has shown adverse effects with reduced patient survival (Hosein et al., 2020). Systemic chemotherapy combinations, including FOLFIRINOX and gemcitabine plus nab-paclitaxel, are currently the mainstay of treatment for patients with advanced pancreatic cancer (Mizrahi et al., 2020). The modified FOLFIRINOX treatment showed promising results with the longest survival reported so far, but it also had high toxicity and adverse events (Tempero, 2019). However, these therapies have limited efficacy and can be associated with significant toxicity. The failure of many clinical trials in pancreatic cancer further emphasizes the urgent need for breakthroughs in this field. This highlights the need for new approaches and strategies to improve the treatment of pancreatic cancer.

Peptide biotherapies have become a significant area of research in the pharmaceutical field, with over 400 peptide drugs in various stages of clinical development and more than 80 approved by the FDA for clinical use (Lee A.C.-L. et al., 2019; Craik and Kan, 2021). While small molecules, proteins, and antibodies are still prevalent in therapeutics, peptides are gaining attention due to their unique biochemical and therapeutic properties. Peptides can be synthesized chemically with reproducibility and modification based on their amino acid composition. They also have high binding affinity, low immunogenicity, and high activity with specific targets (Sun et al., 2018). These features make peptides promising candidates for use in pancreatic cancer applications such as disease diagnosis, molecular imaging, and therapy. Peptides are short chains of amino acids with specific functions determined by their sequence, making them attractive candidates for drug development (Schwieter and Johnston, 2016; Henninot et al., 2018). In the last years, many strategies have been designed to screen for specific amino acid sequences against several target molecules (Andrieu et al., 2019). To identify suitable peptide sequences, many screening strategies have been designed, including high-throughput screening (HTS) techniques such as phage, mRNA/DNA, yeast, ribosomal, and cell display techniques (Dotter et al., 2021; Muttenthaler et al., 2021).

Phage display techniques are powerful tools for screening a variety of suitable peptide ligands with high affinity and selectivity and maximum retention of their biological activity, including disease-specific (Zhang et al., 2022b) or organ-specific peptides (Pleiko et al., 2021), cancer cell/tumor-targeting peptides (Ulfo et al., 2022), peptide-based inhibitors (Miki et al., 2022). This technology is cost-effective and efficient, making it an attractive option for drug discovery and development (Mimmi et al., 2019). In past studies, great efforts have been made to identify a variety of tumor-targeting peptide sequences (Ulfo et al., 2022) and peptide agents that inhibit tumor growth through phage display (Robson and Ghatage, 2011). In this context, our studies have focused on the application of phage display technology in pancreatic cancer therapy, drug delivery, and early detection. Here, we would like to provide a review of recent studies on phage display technology in pancreatic cancer applications. In this review, an overview of the basic biology of phage display technology is provided, including phage vector selection procedures, phage display, and various affinity assessment methods. The article also discusses the challenges associated with the application of phage display in pancreatic cancer and other oncological diseases, as well as future trends and directions. Overall, phage display technology has the potential to significantly impact the development of novel peptide-based therapeutics for pancreatic cancer and other diseases.

### Phage display peptides technology

In 1985, George Smith proposed the phage display technique, which revolutionized the field of biochemistry (Smith, 1985). The technique involves fusing a DNA sequence to a filamentous phage shell protein gene, which displays the peptide on the phage’s surface (Kumar et al., 2019). By doing so, the peptide/protein with the highest binding affinity for a specific target molecule can be selected for isolation (Kehoe and Kay, 2005). This technique has been widely used in the identification and selection of peptides and antibodies for various applications, including cancer treatment and diagnosis. Greg Winter and his colleagues built upon Smith’s technique by creating combinatorial antibody libraries on filamentous phages (McCafferty et al., 1990), leading to the generation of antigen-specific antibodies. For their pioneering work in this field, Smith and Winter shared the Nobel Prize in Chemistry in 2018 (Deutscher, 2019).

### Biology of phagemid vectors

**Figure 2:**
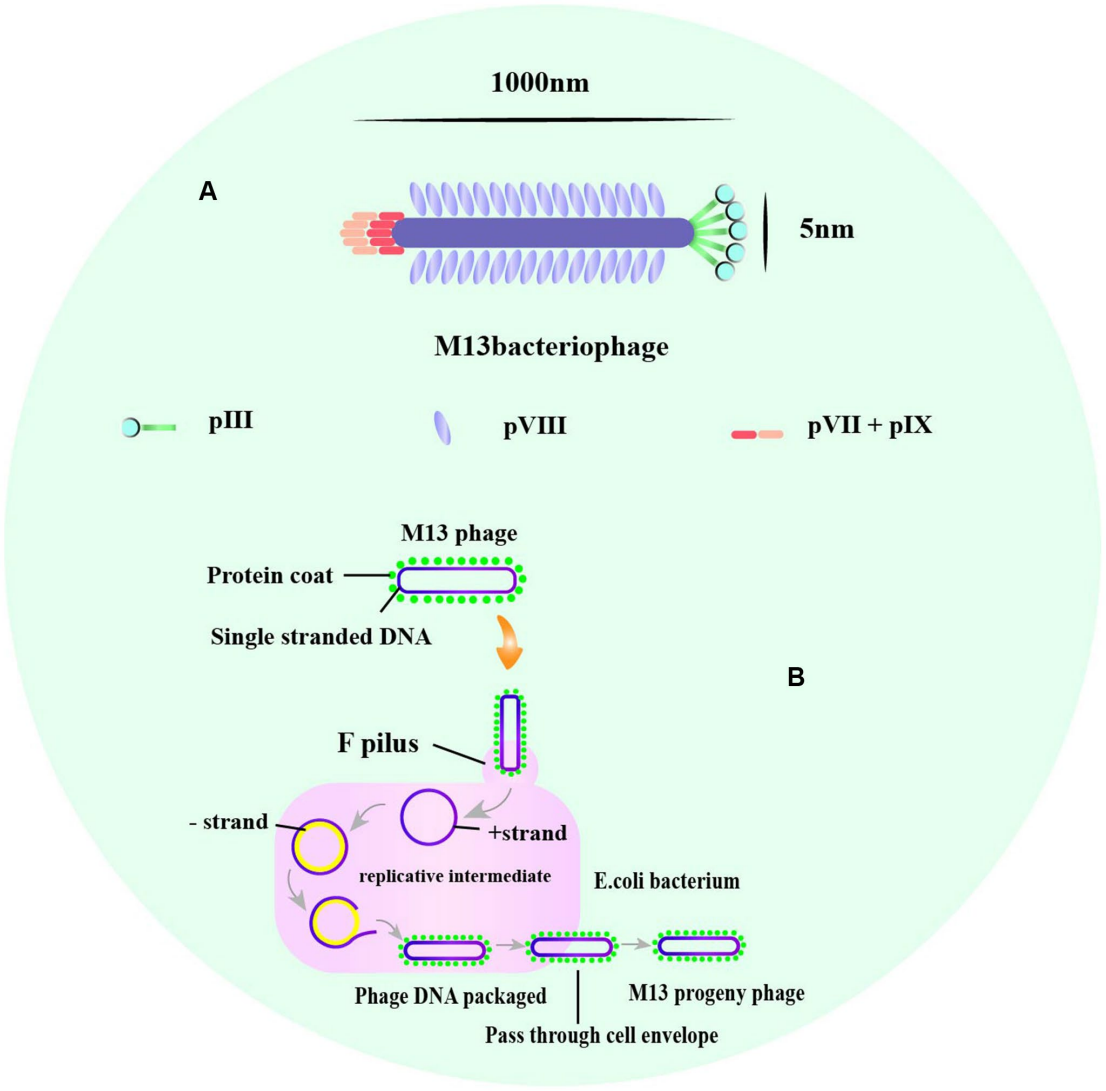
The structure of M13, and the life cycle of phage M13. **(A)** A typical representation of M13 phage with about 1,000 nm in length and 5 nm wide. The major coat proteins are pIII, pVIII, and pVII + pIX complex. **(B)** The M13 life cycle begins with the passage of the phage genome into a host cell mediated by coat protein pIII. The general stages of a viral life cycle include infection, replication of the viral genome, assembly of new viral particles, and release of the progeny [adapted from Saw and Song (2019) and B. Tech. (Biotechnology) III Year Vth Semester EBT-501].

Phage display is a powerful technique used for identifying and selecting peptides with high affinity and specificity for their targets. The technique uses phages, which are viruses that infect bacteria and can be engineered to display a library of amino acid sequences. The most commonly used phage vectors for phage display are M13 and T7 phages. M13 phages replicate in *E. coli* and are relatively simple in structure, making them easy to design to express multiple peptides or proteins (Smith, 2019). The phage has a rod-shaped structure with a length of 1,000 nm and a width of 5 nm (Zhang X. et al., 2022; Figure 2A). It consists of a single-stranded circular DNA genome of approximately 6.4 kb surrounded by five capsid proteins encoded by nine genes (Ch'ng et al., 2022). Among the capsid proteins, pVIII is suitable for peptide and small protein display, while pIII can be used effectively to display large peptides or proteins despite its low protein copy number (Alfaleh et al., 2020; Jaroszewicz et al., 2022).

**Figure 3:**
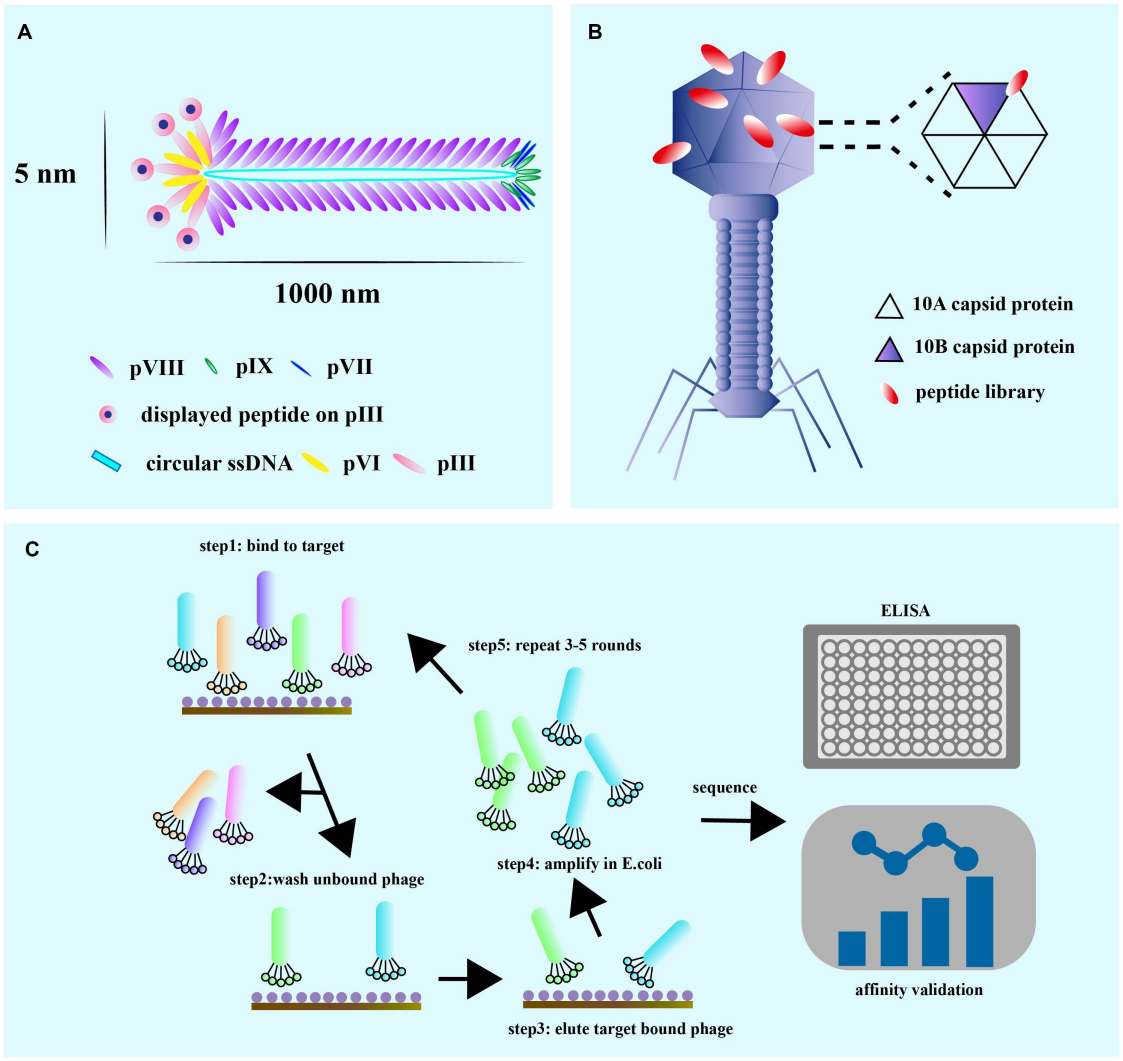
**(A)** M13 is composed of circular single-stranded DNA encapsulated in coat proteins, pVIII, pIX, pVII, pIII, and pVI. The M13 phage coat is primarily assembled from protein p8, but also contains up to five copies each of pIX, pVII, pIII, and pVI. **(B)** The T7 lytic bacteriophage architecture showing copies of the capid protein 10 A and 10B. The gene sequence of the gene engineered peptide can be altered on the capsid 10B. **(C)** The general guidelines for the techniques of phage display and the biopanning selection for high affinity peptides. The biopanning process is used to select high-affinity target-binding peptides from a complex mixture pool of billions of displayed peptides on phage in a combinatorial library. It is commercially available or designed to be used in specialized situations to meet the particular requirements of each experiment [adapted from Zhang et al. (2022)].

M13 and T7 phages are two commonly used phages in biotechnology, with M13 being used for display of peptides on its surface, while T7 is used for overexpression of recombinant proteins. Phage display technology can be used for screening of many enzymes (Aloisio et al., 2021), functional antibody fragments (Bao et al., 2019; Guliy et al., 2023) and inhibitors (Chen S. et al., 2021). Exogenous peptides are displayed on the phage surface and maintain relatively independent spatial conformation and biological activity, facilitating specific recognition and binding of target molecules. M13 phage infection of *E. coli* is a non-lytic process that does not kill the host cell, and phage release from the bacterium requires synergy with the host secretion pathway (Rakonjac et al., 2017; Figure 2B). M13 phage infects *E. coli* in a non-lytic process that does not kill the host cell, and its display of peptides is limited to those that can undergo the host endosomal secretion pathway (Davidson et al., 2020; Figure 3A). On the other hand, T7 phage is released after host cell lysis and can display larger peptides and proteins without the restriction of the host secretion pathway (Piggott and Karuso, 2016; Yue et al., 2022). The T7 phage has an icosahedral head and a short tail (Yu et al., 2022), with the two major capsid proteins, 10A and 10B, protecting phage DNA, and the tail facilitating binding to bacteria (Mahdavi et al., 2022; Figure 3B).

### Phage display peptides biopanning procedure

Biopanning is a phage screening method used to isolate peptide sequences with high affinity and specificity for a target from a large library containing up to 10^13^ phage clones (Davidson et al., 2020). This procedure involves several steps (Figure 3C), starting with the creation of a custom phage library that displays the desired exogenous peptide through the introduction of a exogenous sequence inserted into the phage genome. Next, billions of phages with random display peptides from the library are co-incubated with the target substrate to achieve competitive binding. Weakly bound and unbound phages are then removed by washing buffer, leaving behind phages with high fitness and stronger affinity. The specifically bound phages are eluted and amplified through infection with the host bacterium to create a more selective phage library for the next round of biopanning. This process is repeated for 3–5 rounds, with the titer of eluted phage assessed after each round. ELISA is also used for further affinity assessment of the screened phages. Finally, the exogenous DNA inserted into the phage genome is sequenced after the final round of selection, and the resulting amino acid sequence is a peptide ligand that can bind to the target molecule. Validation of specificity is required to confirm the peptide’s binding ability.

**Figure 4:**
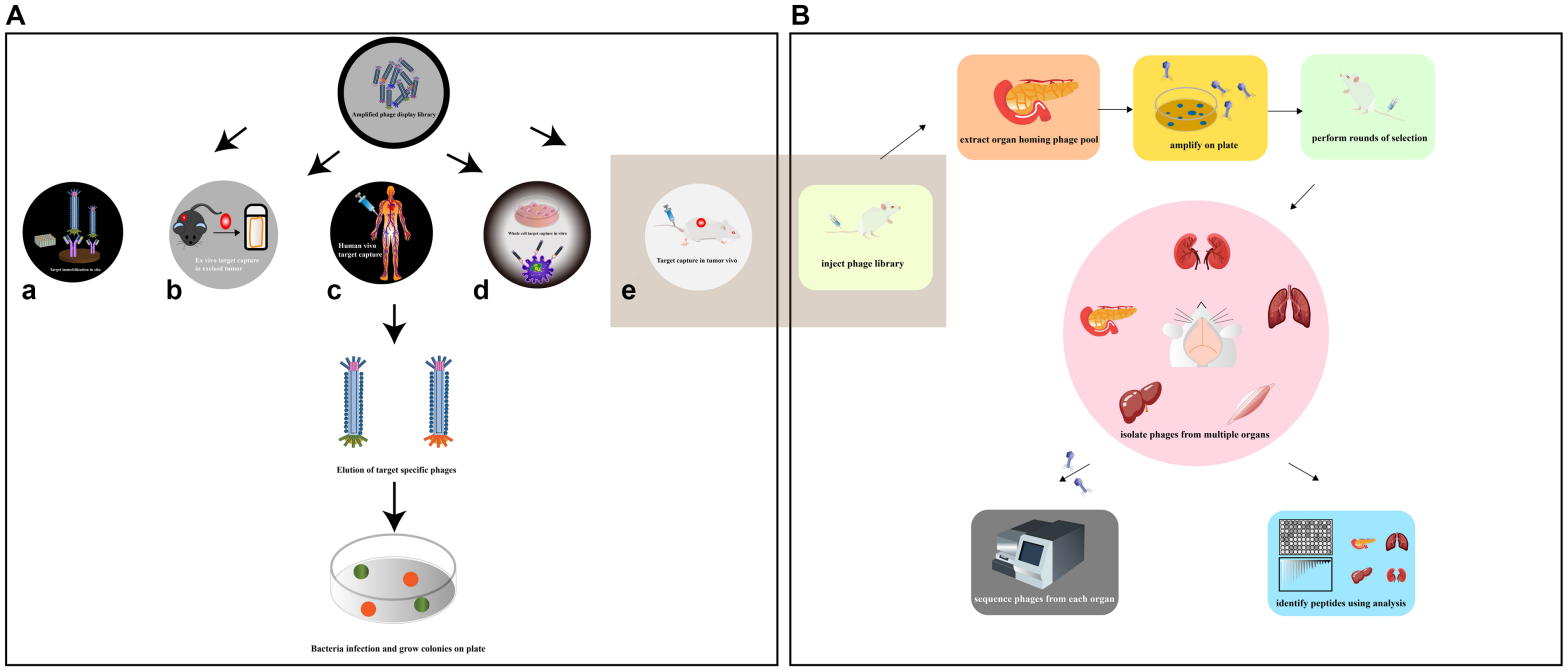
**(A)** Various methods have been employed to collect high-affinity peptides by phage display screening. The situ screening requires that specific targets be coated on plate (a). *Ex vivo* screening should only be applied to select specific rare cells in a heterogeneous population (b). In order to diminish the compatibility of species differences, phage display had been reported to be screened against human patients (c). *In vitro* cell screening is a high-throughput approach for identifying multiple peptides that bind specifically to individual cells and can be performed on adherent cells (d). *In vivo* screening organ-specific peptides can be isolated by biopanning and selection in live animals (e) [adapted from Saw and Song (2019)]. **(B)** Identification of organ-specific peptides using *in vivo* phage display with differential binding analysis [adapted from Pleiko et al. (2021)]. The phage library was injected in mice. The target and control organs are collected and the phage libraries of the target organs are amplified. In the second round, the amplified libraries are injected into mice for further selection of target organs and selective phages. The phage pools isolated from each organ at each round were subjected to high-throughput sequencing (HTS). Differential binding analysis was performed.

Phage screening is a technique used to identify peptides or proteins that can bind to specific targets of interest. Depending on the target being screened, there are several types of phage screening methods, including *in situ* screening, *in vitro* cell screening, *in vivo* animal screening, *ex vivo* screening, and human screening (Figure 4A). *In situ* phage selection is a commonly used method as it allows for easy experimentation on the surface of a well plate or magnetic beads, and can be used to generate functional antibodies that can inhibit cell adhesion (Saw and Song, 2019). However, there is a risk of non-specific binding of isolated peptides during the screening process. In addition, the target is artificially coated onto the plate, which has the potential to alter the actual secondary structure of the target and therefore reduces the usability of the screening peptide (Alfaleh et al., 2020). On the other hand, *in vitro*, *in vivo*, *ex vivo*, and human phage display studies can better mimic the cellular and body conditions and provide more reliable results. Phage *in vitro* cell selection is a method used to identify peptides that bind specifically to individual cells, whether they are cell lines or primary cells (Loi et al., 2013). The protocol has advantages such as preserving the biological function, activity, spatial structure, receptor expression level, and association with neighboring proteins of the target cell. The method can isolate surface-bound and internalized peptides and identify novel cell surface receptors with unknown biological functions. It can also provide information on molecular changes in specific proteins. For instance, Majerova et al. used phage display techniques in a rat primary endothelial cell model to obtain peptides that specifically bind to primary endothelial cells and can traverse the blood–brain barrier (BBB; Majerova et al., 2020). However, unlike *in vitro* conditions, the *in vivo* environment is complex, and *in vivo* studies can better mimic cellular and body primitive conditions.

Phage biopurification in live animals is a technique used to isolate organ/tissue-targeting peptides that more closely resemble physiological conditions. This technique involves introducing peptide phage libraries into live animals via intravenous injection. The phages are then allowed to circulate in the animal, binding to specific organs or tissues. After a period of time, the unbound phages are eluted and those bound to the target organ are collected and amplified by the recipient bacterium ER2738 for the next round of screening. The desired organs/tissues are then collected, homogenized, and the phages are extracted for sequencing (Muttenthaler et al., 2021; André et al., 2022; Figure 4B). This process allows for the identification of specific peptides that target particular organs or tissues *in vivo*. This technique has several advantages over *in vitro* selection methods. First, it allows for the identification of peptides that are more likely to be physiologically relevant, as they have been selected under conditions that more closely resemble the *in vivo* environment. Second, it allows for the identification of peptides that specifically target certain organs or tissues, which can be useful in drug development and diagnostic applications. However, this technique also has some limitations. It can be time-consuming and expensive, and it may not always result in the identification of highly specific or potent peptides. Additionally, the use of live animals raises ethical concerns, and alternative methods such as *ex vivo* organ perfusion systems may be preferable in some cases. Martinez et al. used *in vivo* phage display to identify two HCDR3 peptides that target traumatic brain injury (TBI) sites. The study also revealed novel spatiotemporal TBI targeting motifs and characterized the heterogeneous damage environment (Martinez et al., 2022). This information could have important implications for developing therapies to treat TBI. However, there are some limitations associated with *in vivo* phage display. One of the main drawbacks is the biodegradability of phages by the immune system. The host immune system may recognize and clear the phage particles, limiting the amount of time they can interact with the target tissues. Additionally, one limitation of traditional animal models is the potential differences in peptide binding between species, which can limit the translational potential of identified peptides (André et al., 2022).

**Figure 5:**
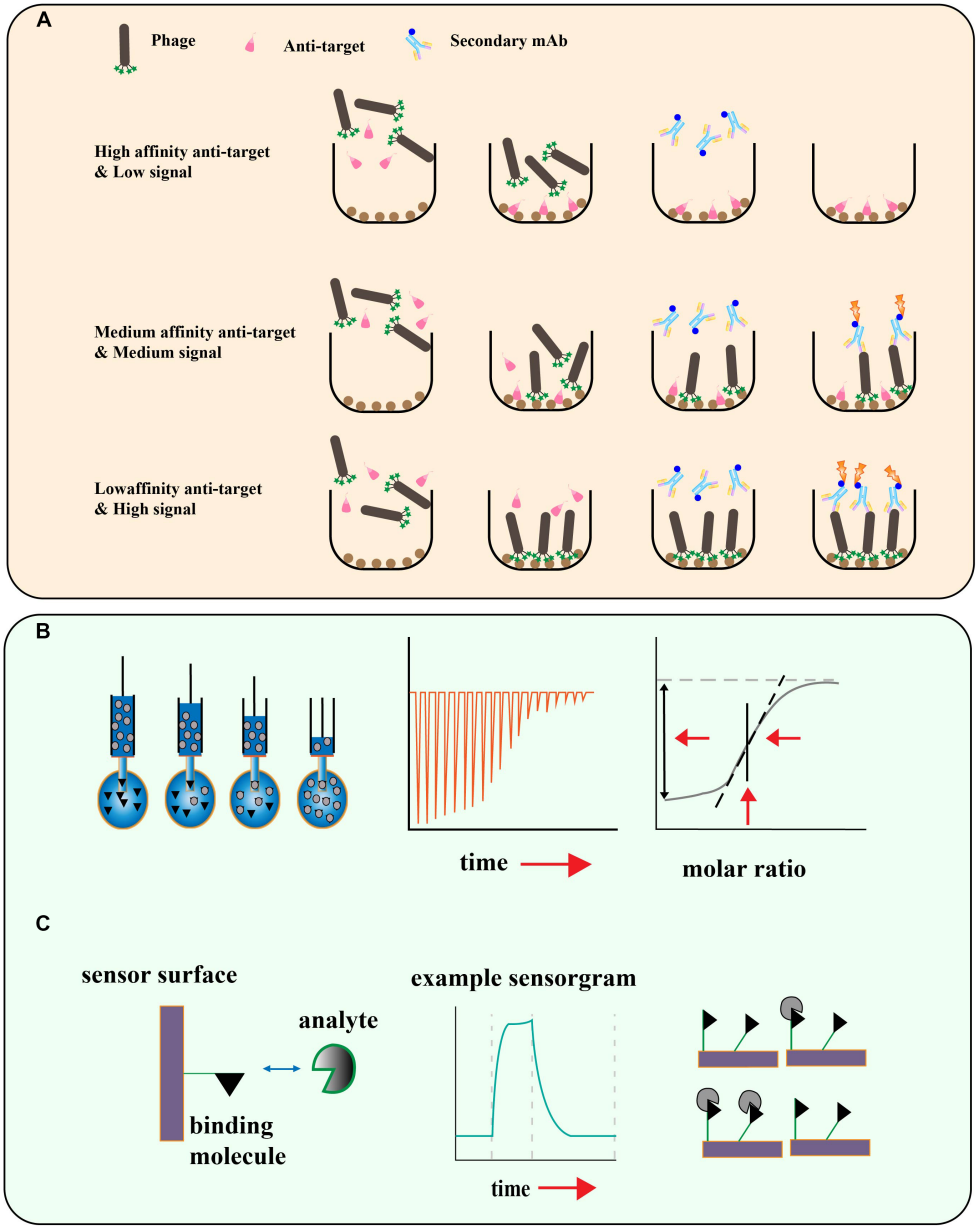
**(A)** Schematic illustration for phage competition assays based on the enzyme linked immunosorbent assay. **(B)** Isothermal titration Calorimetry, titration for measuring thermal capacity change, and calculation of Ka. **(C)** Surface plasmon resonance (SPR) technique and the monitoring of interactions and disassociation of the moving agents [adapted from Laustsen (2016) and Kastritis and Bonvin (2013)].

*Ex vivo* screening is particularly useful for selecting rare cells in heterogeneous populations (Saw and Song, 2019). It involves the use of a phage display library, which contains a large number of different phage particles, each displaying a different peptide or antibody on its surface. The library is incubated with a slide containing the whole cell population, and phages that bind to the rare cells of interest are screened out by protecting them with tiny aluminum disks while non-target cells are cross-linked by UV light. This method enables the selection of ligands that specifically target the rare cells of interest, but it has some limitations. One of the main disadvantages of this method is its low yield compared to other biopanning methods, which means that it may require a large number of phages to achieve adequate selection. Additionally, *ex vivo* screening is limited to the selection of antibodies and is not suitable for peptide selection (Sørensen and Kristensen, 2011). Despite these limitations, the method has been successfully used by Sørensen et al. to select antibodies against rare cells in a heterogeneous cell population (Sørensen and Kristensen, 2011). Phage display screens have been widely used to identify peptides with high affinity and specificity for various targets, including tumors. To overcome this limitation, researchers have developed phage display screens for human patients. Shukla and colleagues demonstrated the feasibility of this approach by injecting phage peptide libraries into patients with malignant tumors prior to surgical tumor removal (Shukla et al., 2013). They were able to recover phages from all tumors and identify multiple tumor-binding phage antibodies, highlighting the potential of phage display screens in human patients for identifying novel diagnostic and therapeutic peptides.

### Phage vectorized co-delivery

Phages are also natural nanocarriers for the delivery of antigenic peptides (i.e., tumor-associated antigenic peptides) and other diagnostic and therapeutic peptides. Phage carriers co-deliver molecules (i.e., immunoreactive lipids, toxins, etc.) capable of exerting antitumor activity. Genes encoding target antigens can be spliced into the phage genome, allowing antigenic peptides to be displayed by fusion into phage capsid proteins. These molecules include immunoreactive lipids, such as polyunsaturated fatty acids, capable of inducing apoptosis or necrosis in tumor cells, and toxins, such as proteins or nucleases, capable of disrupting tumor cell biosynthesis or signal transduction (Fagbohun et al., 2013; Sartorius et al., 2019; Hess and Jewell, 2020). Phages are highly specific and safe, and are able to avoid damage to normal cells. Phages are also able to activate the innate and adaptive immune system, presenting antigens to immune cells in a highly ordered and repetitive manner. For example, a study showed that the presentation of peptide antigens to phage shell proteins via gene fusion significantly inhibited breast cancer growth and prolonged the life span of mice (Arab et al., 2019). Phages are not only able to directly kill tumor-associated bacteria, thereby inhibiting tumor growth and metastasis, but also activate the immune system and enhance the body’s ability to clear tumors. Injection of phages into tumors of mice induces tumor cells to express more antigen-presenting molecules, thus improving the recognition and killing of tumor cells by immune cells (Krut and Bekeredjian, 2018). Combining phage with immune checkpoint inhibitors enhanced the anti-tumor immune response and prolonged the survival of mice (Arab et al., 2019). These results suggest that phages have potential antitumor effects that deserve further exploration and development.

### Bacteriophage T7 for phage display

Both T7 and M13 phage are commonly used as phage display vectors. The T7 phage display system is typically used to display peptide sequences of C-terminal fused 10B shell proteins, while the M13 phage display system is typically used to display peptide sequences of N-terminal fused pIII proteins. The T7 phage display system has less bias relative to the M13 phage display system, while long peptide libraries (12–20mer) are most commonly used to discover cell-binding peptides (Yue et al., 2022). T7 screening libraries can also screen peptides with specific functions from a large number of peptides. In T7 screening libraries, peptides are inserted into T7 genes and then expressed in cells. These peptides can bind to targets and can be used in antitumor therapy (Piggott and Karuso, 2016; Hwang and Myung, 2020). For example, one study found that a T7 screening library could be used to discover peptides with antitumor activity (Sakamoto et al., 2017).

### Evaluation of phage peptides binding affinity

Phage display peptide libraries have shown great potential in identifying ligands specific to a target. However, there is a risk of false-positive results due to the possibility of low affinity peptides being identified during the biopanning process (Bakhshinejad et al., 2016). To ensure the reliability of the screening results, it is crucial to assess the affinity of the identified peptides to their targets. Phage enzyme-linked immunosorbent assays (ELISA) have been developed as a rapid and efficient method to determine if there is an enrichment of phages with high affinity to the target (Panagides et al., 2022; Figure 5A). This technique enables researchers to quickly evaluate the binding specificity and affinity of the identified phage-displayed peptides, and select the most promising candidates for further characterization and development. In this method, the target is encapsulated on a microtitre plate, and purified phage clones are applied at different dilutions. The bound phage is then detected with an HRP-labeled anti-M13 phage antibody, and the final addition of a chromogenic substrate produces a colored product whose absorbance at a specific wavelength is recorded. This allows further screening of individual clones that exhibit the greatest level of enrichment for the true target binding to the antigen. Other methods such as isothermal titration calorimetry (ITC; Chen et al., 2019), surface plasmon resonance (SPR; Zhang et al., 2022a), microscale thermoelectrophoresis (MST; Cho et al., 2022), and Quartz Crystal Microbalance (QCM; Elmlund et al., 2014) can also be used to measure the affinity of the phage display peptide to the target molecule (Figures 5B,C).

**Figure 6:**
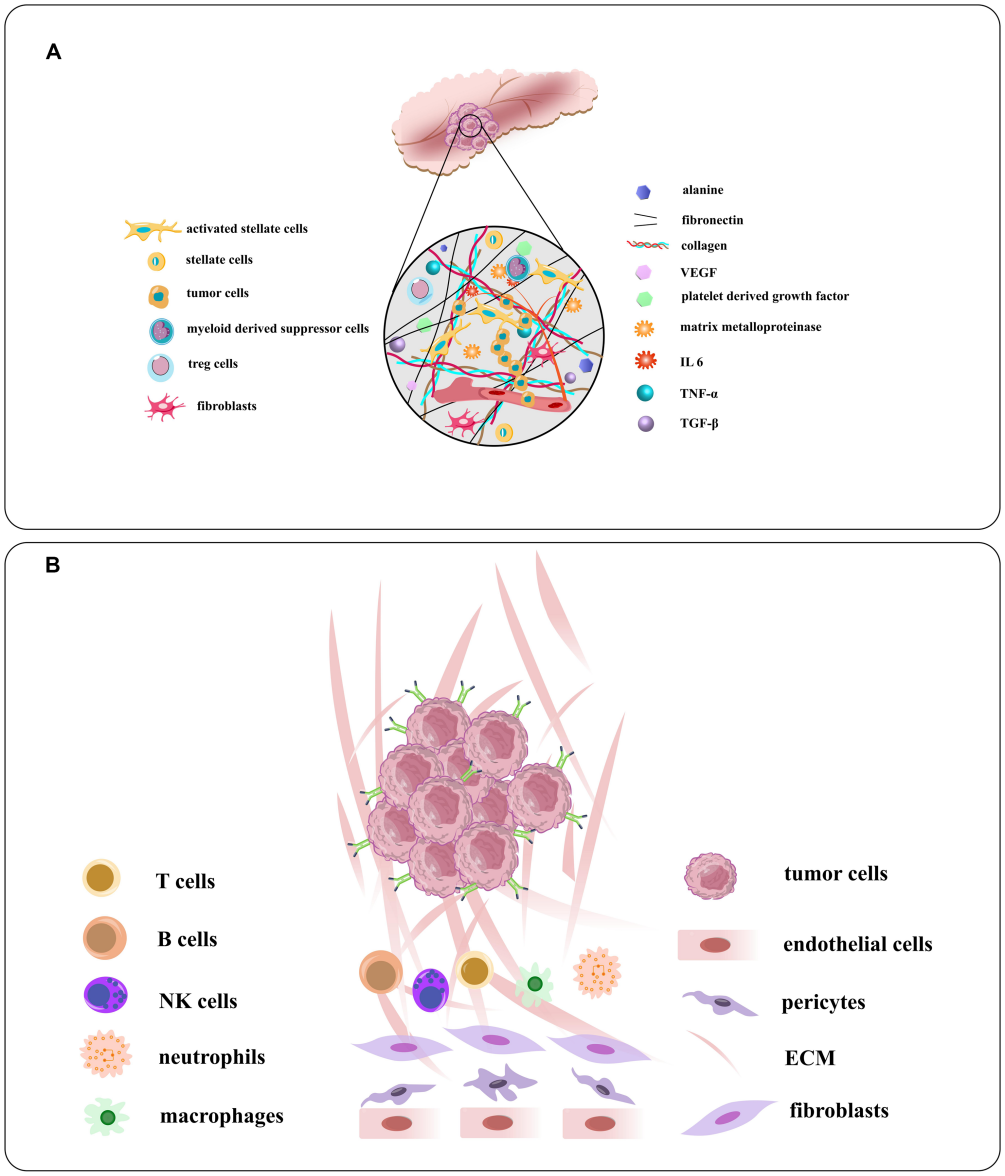
**(A)** Schematic illustration of the TME of PDAC and the densomic stromae that are composed of its densed desmoplastic stroma. Pancreatic stellate cells or PSCs, the principal type of cancer related fibroblast in this condition, make up about half of stroma in all cells, generally active in PDAC. The PSC recruits and secrete extracellular matrix molecules, immunosuppressive and inflammatory cytokines, angiogenesis factors, matrix metalloproteinases, growth factors, and nonessential amino acids. **(B)** The components of the tumor vasculature and the extracellular matrix, tumor stroma cells, and overexpressed receptors on tumor cells [adapted from Verloy et al. (2020) and Saw and Song (2019)].

### Tumor-targeting peptides for pancreatic cancer treatment

Tumor-targeting peptides are short amino acid sequences that can specifically bind to cancer cells and tissues. They have gained interest as a potential tool for cancer diagnosis and treatment due to their low cost, ease of synthesis, and high specificity (Shen et al., 2023). Peptides can be used to target various aspects of the complex environment of pancreatic cancer, including the dysfunctional tumor vascular system, dense extracellular stroma, tumor stromal cells, and overexpressed receptors on tumors (Gutknecht et al., 2017). Phage display biopanning is a commonly used technique to identify peptides that specifically bind to cancer cells. Here, we will discuss some examples of tumor-targeting peptides identified using phage display biopanning and their potential application in the diagnosis and treatment of pancreatic cancer.

### Peptides targeting tumor microenvironment of pancreatic cancer

Pancreatic cancer is a complex disease with a diverse microenvironment consisting of various components such as the vascular system, extracellular matrix, stellate cells, fibroblasts, macrophages, immune cells and cancer cells (Figure 6; Cortesi et al., 2021). As the cancer progresses, these components can transform into a cancer-like phenotype. For instance, most macrophages in pancreatic cancer are M2-like, promoting tumor progression instead of having an anti-cancer M1-like phenotype (Lee C. et al., 2019). By identifying peptides specific to these targets, drugs can be generated to home in on the microenvironment and modulate or disrupt its components (Stopa et al., 2020). These interventions can be classified into targeting the tumor vasculature system, the extracellular matrix, or the tumor stromal cells. Some examples of peptides targeting the TME of pancreatic cancer: iRGD is a peptide that targets integrin αvβ3, which is overexpressed in the vasculature of pancreatic tumors. iRGD can enhance the delivery of co-administered drugs to the tumor parenchyma and increase the efficacy of chemotherapy and immunotherapy in preclinical models of pancreatic cancer (Saifi et al., 2023). Fibroblast activation protein alpha (FAPα) is a marker of cancer-associated fibroblasts (CAFs), which are abundant in the TME of pancreatic cancer. A peptide that targets FAPα can selectively deliver drugs or imaging agents to CAFs and inhibit their tumor-promoting functions (Yuan et al., 2021). Heparan sulfate proteoglycans (HSPGs) are components of the extracellular matrix that play a role in tumor growth and metastasis. A peptide that targets HSPGs can disrupt their interaction with growth factors and prevent the activation of signaling pathways that promote cancer cell proliferation and survival (Li and Kusche-Gullberg, 2016). Neuropilin-1 (NRP-1) is a receptor that is upregulated in pancreatic cancer cells and endothelial cells in the TME. A peptide that targets NRP-1 can inhibit tumor angiogenesis and metastasis by disrupting the interaction of NRP-1 with VEGF and other growth factors. The peptides targeting the TME of pancreatic cancer have the potential to improve treatment outcomes by selectively delivering drugs or imaging agents to specific components of the TME and disrupting key signaling pathways that promote tumor growth and metastasis. However, more research is needed to fully understand the safety and efficacy of these peptides in clinical settings.

**Figure 7:**
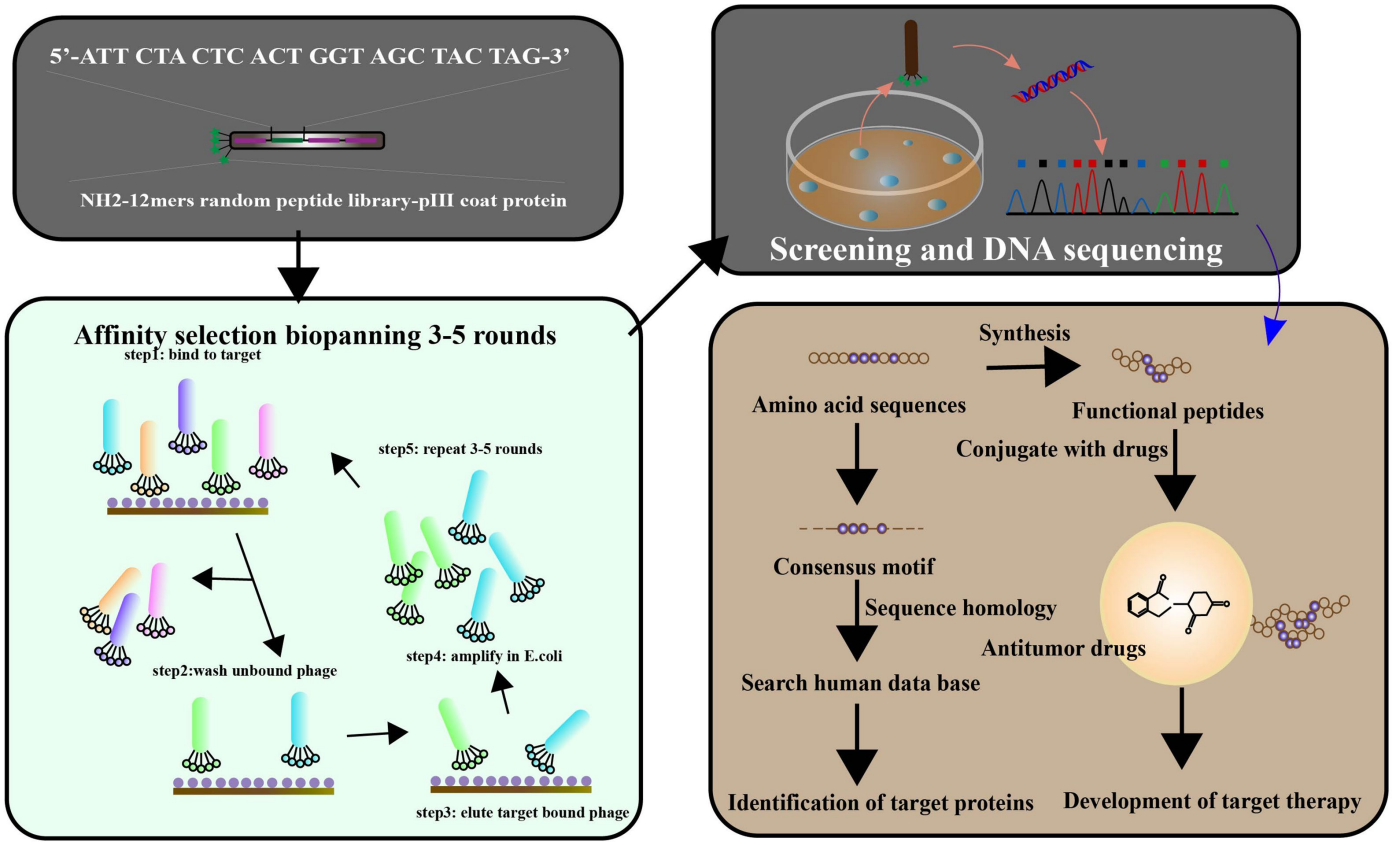
Phage display has been used to identify the ligands targeted to cancer cells. The library of peptides or antibodies can be expressed through the incorporation of the antigen with the etanercept (pIII) of a bacteriophage, resulting in the appearance of the fused protein on virion’s surface. Affinity selection of a phage-displayed peptide library, known as biopanning, represents an excellent method for determining peptide ligands targeting a target of interest. To screen the targets by phage display libraries, and affinity selection against cancer cell lines is carried out pre blankly by normal and cancerous cell lines, respectively. After 3–5 cycles of biopanning, clones for the targeting phages have been chosen. The target ligands were identified and characterized by a synthetic peptide binding and competition assay. Targeting ligands are used to identify cell surface markers, leading to ligand-targeted therapies (adapted from Wu et al. (2006)].

### Peptides targeting the vascular system of pancreatic cancer

Pancreatic cancer is known to be an ischemic tumor; however, angiogenesis plays a crucial role in the growth and progression of pancreatic cancer (Ueda et al., 2022). One potential avenue for therapeutic intervention in pancreatic cancer is the use of peptides that target the vascular system. The vascular system plays a critical role in tumor growth and progression by providing nutrients and oxygen to the cancer cells. Therefore, targeting the vasculature can lead to a reduction in tumor growth and improved patient outcomes. The recruitment of immunosuppressive cells like TAM and MDSC by pancreatic cancer cells in the tumor microenvironment leads to the secretion of pro-angiogenic factors, cytokines, and growth factors that induce angiogenesis in the tumor blood supply and metastasis (Ren et al., 2018). Due to the constant formation of new blood vessels to feed the pancreatic cancer, anti-angiogenic therapy has been found to be effective in treating pancreatic cancer (Zhang et al., 2018). The pancreatic cancer vasculature has a distinct morphology compared to that of normal tissue, which is promoted by peptides that promote pericyte coverage and lead to the normalization of the vascular system (Valetti et al., 2014). NGR (Asn-Gly-Arg), which targets aminopeptidase N/CD13 expressed on tumor endothelial cells. NGR peptides conjugated with chemotherapeutic agents have demonstrated efficacy in preclinical models of pancreatic cancer (Seidi et al., 2018). There is a peptide called F3, which binds to nucleolin, a protein expressed on the surface of tumor endothelial cells. F3 has been shown to inhibit angiogenesis and tumor growth in preclinical models of pancreatic cancer (Romano et al., 2019). The peptides targeting the vascular system in pancreatic cancer offer a promising avenue for therapeutic intervention. The above-mentioned peptides and their potential targets can be used to develop more effective treatments for this deadly disease.

### Peptides targeting matrix metalloproteinases of pancreatic cancer

Peptides targeting matrix metalloproteinases (MMPs) have been investigated as a potential therapeutic strategy for pancreatic cancer, which is known for its aggressive nature and poor prognosis. MMPs are a family of enzymes that play a key role in the degradation, dynamic remodeling and turnover of extracellular matrix (Jones et al., 1999), and are upregulated in many types of cancer, including pancreatic cancer. By targeting MMPs, it may be possible to inhibit tumor invasion and metastasis. Pancreatic cancer is characterized by upregulation of MMPs in the microenvironment, which play a key role in angiogenesis and metastasis (Cox, 2021). Higher expression of specific MMPs has been associated with increased invasion and metastasis of pancreatic cancer (Ho et al., 2020). Phage display screening have been used to identify various peptides that can inhibit the activity of the MMP family, providing a potential therapeutic approach to target this pathway in pancreatic cancer [Figure 7; Maola et al., 2019). Several studies have investigated the use of peptides targeting MMPs in pancreatic cancer. Lu et al. (2012)] identified novel peptides for MMP-2 inhibition, which is important for inhibiting tumor growth based on the principle of passive targeting of specific features of the tumor vascular system. A peptide called CTTHWGFTLC, which targets MMP-2, has been shown to inhibit pancreatic cancer cell invasion and migration *in vitro* (in cell culture) and *in vivo* (in animal models; Gasparini-Junior et al., 2019; Tarazona et al., 2022). Similarly, a peptide called DFKDKFVQWY, which targets MMP-9, has been shown to inhibit pancreatic cancer cell proliferation and invasion *in vitro* and *in vivo* (Grünwald et al., 2016a,b). Other peptides targeting MMPs that have been investigated for pancreatic cancer include: PEX, a peptide that targets both MMP-2 and MMP-9, has been shown to inhibit pancreatic cancer cell invasion and metastasis *in vivo* (Knapinska et al., 2017; Quintero-Fabián et al., 2019). CGKRK, a peptide that targets MMP-14, has been shown to inhibit pancreatic cancer cell invasion and migration *in vitro* and *in vivo* (Li M. et al., 2021).

**Figure 8:**
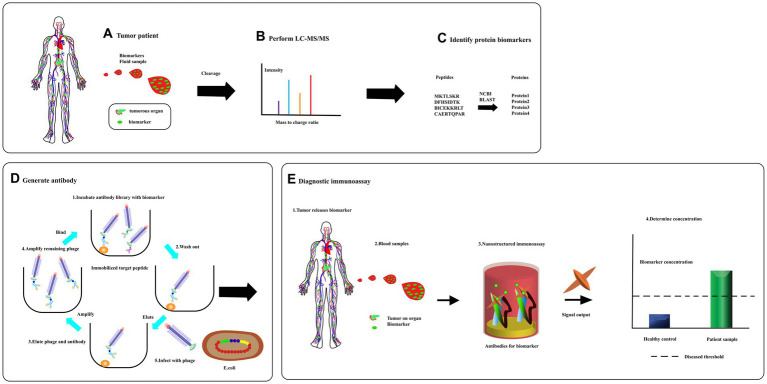
From biomarker discovery to diagnosis. **(A)** Specific proteins are released to circulate in response to the tumors. **(B)** A sample is then separate using liquid chromatography and fractions analyzed using tandem mass spectrometry. **(C)** Database is searched for full-length protein sequence using an amino acid sequence through bioinformatics program like BLAST. **(D)** A peptide complete, or a synthetic, peptide is used in a phage display to produce a specific recombinant antibody. **(E)** Serum was detected for diagnosis [adapted from Kierny et al. (2012)].

### Peptides targeting tumor-associated macrophages of pancreatic cancer

Pancreatic cancer is a deadly disease with a low survival rate, and one of the reasons for this is the presence of tumor-associated macrophages (TAM) within the tumor microenvironment. TAMs are immune cells that infiltrate the tumor and play a critical role in promoting tumor growth, invasion, and metastasis. Therefore, targeting TAMs is a promising strategy for the treatment of pancreatic cancer. In pancreatic cancer, high densities of TAMs have been linked to poor prognosis, and these TAMs express an M2-like phenotype (Liu et al., 2013, 2022). Therefore, targeting M2-like TAMs has been explored as a potential therapeutic strategy, and selective depletion of this macrophage subpopulation has shown positive results (Yu et al., 2019; Dallavalasa et al., 2021). Phage display technology can be used to screen for peptides that specifically bind to M2-like TAMs, which can then be developed for clinical application. These peptides can potentially be used to deliver therapeutic agents specifically to M2-like TAMs or to induce their depletion, leading to improved treatment outcomes in pancreatic cancer. Fernanda I Staquicini and colleagues discovered a cyclic peptide called CSSTRESAC that binds to a protein receptor called protein disulfide isomerase A3 (PDIA3), which is specifically expressed on the surface of M2-like tumor-associated macrophages (TAM). In various mouse models of breast cancer, including TNBC and non-TNBC, systemic administration of CSSTRESAC led to a shift in cytokine profile toward an anti-tumor immune response and delayed tumor growth. Additionally, CSSTRESAC was shown to enable targeted delivery of therapeutic diagnosis to tumors using digital models (Staquicini et al., 2021). The study suggests that CSSTRESAC may be a promising therapeutic option for breast cancer treatment. A peptide called CLT1 has been shown to selectively bind to TAMs and inhibit their function, leading to a reduction in tumor growth and metastasis in preclinical models of pancreatic cancer (Valilou et al., 2018; Xia et al., 2022). Another peptide called FBP has been shown to target both TAMs and cancer cells in pancreatic cancer (Liu et al., 2021). FBP binds to a protein called fibroblast activation protein (FAP), which is expressed on both TAMs and cancer cells. By targeting FAP, FBP can inhibit the function of both TAMs and cancer cells, leading to a reduction in tumor growth and metastasis (Valilou et al., 2018). Other peptides targeting TAMs in pancreatic cancer include CCL2 inhibitors, which block the recruitment of TAMs into the tumor microenvironment, and CSF-1R inhibitors, which block the activation of TAMs (Monti et al., 2003; Ho et al., 2020; Hourani et al., 2021; Zhu et al., 2021).

### Peptides that bind intracellular targets of pancreatic cancer

Intracellular targets are molecules that are located inside cells and are the targets of drugs or other therapeutic agents. These targets include proteins, nucleic acids, and other molecules that play important roles in cellular processes such as signaling pathways and gene expression. Peptides that can target intracellular checkpoints in tumor cells have been explored as a potential therapeutic strategy (Zhang M. et al., 2022). These peptides are able to bind to and inactivate targets within the cell, such as transcription factors, enzymes, or overexpressed oncogenes (Elsadek and Hassan, 2021; Yi et al., 2021; Wang et al., 2022). Oncogene-targeted therapeutic strategies have been shown to sensitize tumor cells to the effects of chemotherapy and radiotherapy, acting synergistically with conventional treatments (Yang et al., 2013; McNerney et al., 2022). There are several peptides that have shown promising results in targeting intracellular pathways in pancreatic cancer cells. Antennapedia peptide is a cell-penetrating peptide that can cross the cell membrane and target intracellular pathways. Studies have shown that antennapedia peptide can be used to deliver various therapeutic molecules into cells and inhibit tumor growth (Fujimoto et al., 2000; Hosotani et al., 2002; Marqus et al., 2017). Antennapedia peptide can enter cells by interacting with the plasma membrane and then translocating across the membrane into the cytoplasm. Once inside the cell, it can target intracellular pathways (Justine and Jean, 2019). iRGD peptide can target tumor cells by binding to αv integrins that are overexpressed in pancreatic cancer. iRGD peptide can also penetrate the tumor tissue and reach the cancer cells, making it a promising candidate for drug delivery (Lo et al., 2018; Hurtado de Mendoza et al., 2021). LyP-1 peptide targets the p32 protein, which is overexpressed in pancreatic cancer cells. Studies have shown that LyP-1 peptide can deliver drugs to pancreatic cancer cells and inhibit tumor growth (Lin et al., 2020). PTD-DRBD peptide targets the RNA-binding protein HuR, which is overexpressed in pancreatic cancer cells. PTD-DRBD peptide can inhibit the activity of HuR and induce apoptosis in pancreatic cancer cells (Eguchi et al., 2009).

### Pancreatic cancer therapeutic evaluation methods for peptides

The selection of high-throughput phage peptide libraries allows the identification of many potential candidate peptides, and various experimental approaches are used to assess the specificity and therapeutic efficiency of these peptides for pancreatic cancer applications. When evaluating the therapeutic potential of peptides for pancreatic cancer, there are several methods that can be used.

Cell viability assays measure the ability of pancreatic cancer cells to survive and grow in the presence of the peptide. Various methods such as MTT assay, cell proliferation assay, or colony formation assay can be used to assess the efficacy of the peptide (Eguchi et al., 2009). Apoptosis assays evaluate the ability of the peptide to induce programmed cell death in pancreatic cancer cells. Methods such as flow cytometry, Annexin V staining, or TUNEL assay can be used to detect apoptosis (Pfeifer et al., 2021). Animal models of pancreatic cancer can be used to evaluate the efficacy of the peptide in inhibiting tumor growth and metastasis. Tumor volume measurement, histological analysis, or imaging techniques such as PET-CT can be used to monitor tumor progression (Pan et al., 2016). Peptides can be conjugated with chemotherapeutic drugs or imaging agents to evaluate their ability to deliver therapeutic molecules to pancreatic cancer cells. Methods such as fluorescence microscopy, confocal imaging, or radioimaging can be used to visualize the peptide-drug conjugate *in vitro* or *in vivo* (Kimura et al., 2022). Peptides can target specific intracellular pathways or proteins in pancreatic cancer cells. Therefore, mechanistic studies can be performed to elucidate the underlying mechanisms of the peptide’s anti-tumor activity. Methods such as Western blot, RT-qPCR, or gene silencing can be used to study the effects of the peptide on specific signaling pathways or gene expression (Asar et al., 2020). These methods can be used individually or in combination to comprehensively evaluate the therapeutic potential of peptides for pancreatic cancer. Peptide inhibition of pancreatic cancer-associated proteins *in vitro* is a prerequisite for their ability to operate in more complex cellular environments and *in vivo*. Levente ED (Dókus et al., 2020) et al. constructed novel drug-peptide couples based on the principle of specific binding of antitumor drugs to molecular targets for targeted treatment of pancreatic cancer. Stability and metabolism of fusion peptides can be accomplished by liquid chromatography-mass spectrometry (LC–MS; Dókus et al., 2020). *In vitro* cell invasion assays can be determined by matrix gel assays (Hall and Brooks, 2001). These approaches include *in vitro* inhibition assays, stability and metabolism analysis, *in vitro* cell invasion assays, and fluorescent labeling complexes for sentinel and *in vitro* monitoring (Hall and Brooks, 2001). These methods are crucial for determining the potential of phage display-derived peptides to be used in more complex cellular environments and *in vivo* for the treatment of pancreatic cancer. At the cellular level, biocompatibility, cell membrane penetration, and cytotoxicity must be assessed (Joyce et al., 2003). In animal models of pancreatic cancer, the therapeutic efficacy of these peptides can be evaluated in more realistic physiological conditions. Tissue staining and immunoblot analysis are classical measures of assessment for the evaluation of peptide functions (Zheng et al., 2020). The extensive *in vitro* and *in vivo* exploration of phage display peptide functions is necessary to ensure their safety and efficacy before they can be developed as drugs.

### Phage display peptides for early diagnosis of pancreatic cancer

Pancreatic cancer is a highly lethal cancer that often goes undetected until its advanced stages, making early detection and diagnosis crucial for improving patient outcomes. Phage display technology is a powerful tool for the early diagnosis of pancreatic cancer. Here are a few ways that phage display technology can be used for the early diagnosis of pancreatic cancer.

### Identification of pancreatic cancer-specific biomarkers

Phage display libraries can be screened against pancreatic cancer cells or tissue samples to identify peptides that bind specifically to tumor cells or tumor-associated biomarkers. These peptides can be used as diagnostic tools to detect the presence of pancreatic cancer cells in blood or other bodily fluids. Phage display technology has been utilized to identify accurate, low-cost, and less invasive pancreatic cancer-specific biomarkers. The membrane linker A2 (ANXA2) gene has been identified as strongly associated with pancreatic adenocarcinoma (PAAD; Karabulut et al., 2020). Ma et al. recently reported a near-infrared fluorescence (NIRF) probe for the immediate detection of PAAD (Ma et al., 2022). They utilized the PH.D-7 phage display library to screen for candidate peptides that were sensitive to ANXA2 but not to GST. Through three rounds of phage display screening, a protein YW7 specifically targeting ANXA2 was identified. The peptide FITC-YW7 was synthesized and labeled with a fluorescent dye to create a fluorescent probe. The FITC-YW7 probe demonstrated high sensitivity and accuracy in real experiments and has potential applications in clinical diagnosis and real-time monitoring of PAAD. In 2020, Mallika C. Asar et al. conducted a study to identify functional peptides for the early diagnosis of pancreatic cancer using peptide binding tests (Asar et al., 2020). The study was designed in two phases: a discovery phase and a validation phase. In the discovery phase, researchers used phage display to screen candidate peptides from immortalized human pancreatic cells and human pancreatic cancer cells, and further evaluated the specificity of the selected peptides by ELISA and peptide competition binding assays. The results showed that MCA1 and MCA2 had high specific affinity for human pancreatic cancer cells (Mia Paca-2) and no significant binding to immortalized human pancreatic cells (hTERT-HPNE), demonstrating their specificity for Mia Paca-2 cells. In the validation phase, the peptides were tested against other cancer cell lines, including human prostate cancer cell line (LNCaP), human embryonic kidney cell line (HEK 293) and human ovarian cancer cells (SK-OV-3). The results showed no significant binding of MCA1 and MCA2 to these cell lines, suggesting that these peptides can distinguish pancreatic cancer cell lines from normal pancreatic tissue cell lines and other tumor cell lines. The combination of MCA1 and MCA2 may provide a new blood biomarker test for the early and accurate diagnosis of pancreatic cancer.

Autoantibodies are antibodies produced by the body’s immune system in response to abnormal proteins, which can be useful as diagnostic tools for blood-based biomarkers due to their stability and specificity (Sexauer et al., 2022). In the case of pancreatic cancer, autoantibodies may be triggered by pancreatic cancer-specific target proteins (Capello et al., 2015). Phage display integrated protein microarrays have been used to screen for pancreatic cancer autoantibodies as blood-based biomarkers for early diagnosis (Bloch et al., 2008). Similarly, Cleutjens et al. utilized peptide array technology to develop antibody biomarkers for ruptured atherosclerotic lesions. Phage display libraries were prepared using mRNA from ruptured peripheral human atherosclerotic plaques, and sera from patients with ruptured atherosclerotic lesions were screened for phages containing immunoreactive peptides. Antibodies reacting to both E2 and E1 peptides were found to be particularly sensitive for the early diagnosis of acute myocardial infarction. Future studies should include a sufficient number of patients seen very early after the onset of symptoms, as well as additional control patient groups, to compare the utility of these biomarkers with those currently in clinical use (Cleutjens et al., 2008). The use of autoantibodies and peptide arrays as potential blood-based biomarkers holds promise for the early and accurate diagnosis of various diseases, including pancreatic cancer and acute myocardial infarction.

### Molecular imaging agents

Peptides identified through phage display can be conjugated to imaging agents, such as fluorescent dyes or radioactive tracers, to visualize the location and extent of pancreatic tumors. These imaging agents can be used to guide biopsy procedures or monitor tumor growth and response to treatment. Phage display-derived peptides have also been utilized for *in vivo* molecular imaging as specific contrast agents, which could potentially improve the sensitivity of early cancer detection. The human gastric mucin MUC5AC, which is a specific marker for precancerous lesions in the colon, has been used as a target for molecular imaging. Yannick-Rosses et al. demonstrated the detection of MUC5AC accumulation in xenografts and mouse stomachs using magnetic resonance imaging (MRI) with ultra-small iron oxide particles coupled to disulfide-bound heptapeptides (USPIO). The heptapeptides were identified using a screening phage display, and positive selection was performed on purified MUC5AC from fresh human colonic adenomas, while negative selection was performed on purified human MUC2, which is predominantly present in normal colorectal tissue. The MUC5AC-USPIO contrast agent was found to be specific for cancer cell lines capable of secreting MUC5AC, and it was not detected in cell lines unable to secrete MUC5AC. The potential use of MUC5AC contrast agents in diagnosis, early detection of colorectal cancer recurrence after treatment, and in mechanistic studies involving MUC5AC has been highlighted by this study (Rossez et al., 2016).

### Blood-based tests for the early diagnosis of pancreatic cancer

After surgical resection of pancreatic tumors, there is a high risk of tumor recurrence. Phage display technology can be used to identify peptides that bind specifically to residual cancer cells or tumor-associated biomarkers in blood or other bodily fluids, enabling early detection of recurrent disease. Pancreatic cancer has a poor prognosis mainly due to the delayed diagnosis of the disease. Early and accurate diagnosis of pancreatic cancer is crucial, especially with the advent of disease-modifying therapies. Blood-based tests for the early diagnosis of pancreatic cancer offer a cheaper, less invasive, and more accessible alternative. Phage display technology has been utilized to screen for functional peptides or antibodies from the blood of pancreatic cancer patients and healthy individuals to develop blood-based biomarker detection tests based on these phage-derived peptides (Figure 8). This approach has provided new insights into the early diagnosis of pancreatic cancer. Furthermore, new biomarkers for pancreatic cancer diagnosis, such as microRNA (Zhang W. et al., 2022), have been emerging in recent years. Phage display technologies can be used to develop high-affinity, high-sensitivity probes that can be combined with various biosensors for rapid blood testing, showing great potential in the development of more effective diagnostic tools for pancreatic cancer.

### Challenges of phage display strategies in pancreatic cancer applications

Peptides can offer several advantages as targets for biomedical applications, including high specificity, low toxicity, small size, and high penetration (Brissette et al., 2006; Scodeller and Asciutto, 2020; Li C. M. et al., 2021). However, developing these peptides for clinical use can be challenging due to their lack of specificity in their linear conformation (Ganjiwale and Cowsik, 2013). To overcome this issue, researchers have decorated the surface of nanocarriers with short linear peptides to increase the probability of interaction with specific ligands. Yongmei Wu et al. developed a nanocarrier system by coupling short peptides to thrombin-binding aptamers (TBA) and bound hemoglobin to hybrids to prepare hemoglobin/G4 peptides. They combined porous platinum nanotubes (PtNTs) with reduced graphene oxide (rGO) to develop an electrochemical aptamer sensing system with extremely low detection limits for the quantification of low-level biomarkers. PtNTs@rGO provided a large surface area for anchoring abundant hemoglobin/G4-peptide hybrids via Pt-NH affinity, leading to the formation of hemoglobin/G4-peptide-PtNTs@rGO biocouples. PtNTs@rGO with large surface area act as excellent nanocarriers for highly loaded heme/G4-peptide hybrids, leading to the formation of heme/G4-peptide PtNTs@rGO biocouples as secondary aptamers and further enhancing the signal. This electrochemical aptamer sensing system has extremely low detection limits and can be used for the quantification of low-level biomarkers (Wu et al., 2017). Another example of using nanocarriers with modified peptides is by Chen et al. who modified viral proteins onto the surface of albumin nanoparticles (Alb NPs) loaded with fluorescent dyes. This modification enabled the recognition and binding of target miRNA double-stranded bodies, allowing for rapid and sensitive quantification that can significantly advance the use of miRNAs as biomarkers in clinical practice (Wei et al., 2017).

Peptides with 5 or fewer amino acids are usually water soluble and their solubility decreases with the length of the peptide. However, peptides screened by phage display biopanning range from 7 to 30 amino acids. In aqueous solution, these peptides can conform to a specific 3-dimensional structure that allows specific binding to their receptors. The longer peptides screened by phage display biopanning can form specific 3D structures that allow them to bind to their receptors. Improper solubilization can lead to loss of peptide activity. Phage display biopanning has generated numerous high-affinity and highly specific peptide ligands for pancreatic cancer therapy. These peptide-based ligands have shown promising results in enhancing pancreatic cancer therapy, such as increased tumor accumulation (Rana et al., 2012), specific tumor targeting (Huang et al., 2022), and improved tumor suppression when coupled to anti-cancer drugs (Dókus et al., 2020). However, for successful translation into the clinic, peptide-targeted ligands need to be optimized for their affinity, water solubility, and specificity. Advances in technology allow the use of primary phage display screening and secondary computational optimization methods to develop optimal peptides for targeting any receptor of interest in pancreatic cancer therapy.

The development of peptide-based biotherapeutics has shown great promise in pancreatic cancer diagnosis, treatment, and drug delivery due to their small size, low cost, and high specificity (Table 1). Phage display screening has been a powerful tool in identifying functional peptides for these applications. However, there are still challenges that need to be addressed to accelerate their clinical application. For example, the low stability and bioavailability of peptides can limit their effectiveness. In addition, the development of peptide-based drug delivery systems requires careful consideration of factors such as pharmacokinetics, biodistribution, and toxicity. Therefore, further research is needed to optimize peptide-based biotherapeutics for clinical use, and to overcome these challenges in order to realize the full potential of this promising technology in the fight against pancreatic cancer. Chemically synthesized peptides may not maintain the original conformation of the phage tip display, and multiple copies of exogenous display peptides on the phage result in strong binding to the target during selection. One major issue is the maintenance of the original conformation of phage-derived peptides during chemical synthesis, as well as ensuring that single synthetic peptides are strong enough to bind to the target. Sequence analysis and structural prediction of phage-derived peptides must also be carefully considered, as well as the possibility of supramolecular assembly of the peptide (Gray et al., 2022; Zhou et al., 2023). False positives are also a challenge in phage display screening (Bakhshinejad et al., 2016), with many phages already occupied by non-specific adsorption before positive selection. Considering that phages bind to plastic substrates or sealers (Lamboy et al., 2009), a large number of phages are already occupied by non-specific adsorption prior to positive selection. Additionally, amplification is necessary for the enrichment of eluted phages, but it increases the risk of introducing biological bias and reducing library diversity. For example, some phages cannot be preferentially amplified due to poor infectivity, while wild-type phages introduced due to contamination are preferentially amplified.

**Table 1:** The various types of peptides used for diagnostic and therapeutic purposes.

Sequence	Target molecules	Target molecule location	Role	Type	References
iRGD	integrin αvβ3	Intra-tumoral environment	Enhances the delivery of co-administered drugs to the tumor parenchyma and improves the efficacy of chemotherapy and immunotherapy	Therapeutic peptides	Saifi et al. (2023)
ENO1	Fibroblast activation protein alpha (FAPα)	Intra-tumoral environment	Can selectively deliver drugs or imaging agents to cancer-associated fibroblasts and inhibit their pro-tumor function	Therapeutic peptides	Yuan et al. (2021)
OC-46F2	Heparan sulfate proteoglycans (HSPGs)	Intra-tumoral environment	Can disrupt its interaction with growth factors and inhibit vascular maturation and tumor growth	Therapeutic peptides	Li and Kusche-Gullberg (2016), Onyeisi et al. (2020)
KDKPPR	Neuropilin-1 (NRP-1)	Intra-tumoral environment	Tumor angiogenesis and metastasis can be inhibited by disrupting the interaction of NRP-1 with growth factors such as VEGF.	Therapeutic peptides	Gries et al. (2020)
NGR peptideGNGRAHA	CD13,α_v_β_3_	Intra-tumoral environment	Selectively binds to tumor endothelial cells, induces thrombosis and causes tumor infarction	Therapeutic peptides	Seidi et al. (2018)
F3	A protein nucleoprotein expressed on the surface of tumor endothelial cells	Vascular system	May inhibit angiogenesis and tumor growth	Therapeutic peptides	Romano et al. (2019)
CTTHWGFRLC	MMP-2	Targeting matrix metalloproteinases in pancreatic cancer (MMPs)	Inhibition of pancreatic cancer cell invasion and migration *in vitro* (cell culture) and *in vivo* (animal models)	Therapeutic peptides	Gasparini-Junior et al. (2019), Tarazona et al. (2022)
DFKDKFVQWY	MMP-9	Targeting matrix metalloproteinases in pancreatic cancer (MMPs)	Inhibits the proliferation and invasion of pancreatic cancer cells *in vitro* and *in vivo*	Therapeutic peptides	(Grünwald et al., 2016a,b)
PEX	MMP-2和MMP-9	Targeting matrix metalloproteinases in pancreatic cancer (MMPs)	Proven to inhibit *in vivo* invasion and metastasis of pancreatic cancer cells	Therapeutic peptides	Knapinska et al. (2017), Quintero-Fabián et al. (2019)
CGKRK	MMP-14	Targeting matrix metalloproteinases in pancreatic cancer (MMPs)	Inhibits the invasion and migration of pancreatic cancer cells *in vitro* and *in vivo*	Therapeutic peptides	Li C. M. et al. (2021), Li M. et al. (2021)
CSSTRESAC	Disulfide isomeraseA3 (PDIA3)	Tumor-associated macrophages	Leads to a shift in cytokine profile toward an anti-tumor immune response and delays tumor growth; demonstrates the ability to deliver therapeutic diagnostic targeting to tumors using digital models	Therapeutic peptides	Staquicini et al. (2021)
CLT1	TAMs	Tumor-associated macrophages	Leading to reduced tumor growth and metastasis in preclinical models of pancreatic cancer	Therapeutic peptides	Valilou et al. (2018), Xia et al. (2022)
FBP	Fibroblast activator protein (FAP)	Tumor-associated macrophages	Inhibits the function of TAMs and cancer cells, thereby reducing tumor growth and metastasis	Therapeutic peptides	Valilou et al. (2018)
CCL2 inhibitors/CSF-1R inhibitors	TAMs	Tumor-associated macrophages	Blocking recruitment/activation of TAM into the tumor microenvironment	Therapeutic peptides	Ho et al. (2020), Hourani et al. (2021), Monti et al. (2003), Valilou et al. (2018), Zhu et al. (2021)
LyP-1	p32	Targeting of intracellular peptides	Can deliver drugs to pancreatic cancer cells to inhibit tumor growth	Therapeutic peptides	Lin et al. (2020)
PTD-DRBD	The RNA-binding protein HuR	Targeting of intracellular peptides	Inhibits HuR activity and induces apoptosis in pancreatic cancer cells	Therapeutic peptides	Eguchi et al. (2009)
YW7	Membrane linker A2 gene (ANXA2)	Target-specific biomarkers	Potential applications in clinical diagnosis and real-time monitoring of pancreatic adenocarcinoma	Diagnostic peptides	Ma et al. (2022)
MCA1 and MCA2	Mia Paca-2	Target-specific biomarkers	Combined test may offer a new blood biomarker test for the early and accurate diagnosis of pancreatic cancer	Diagnostic peptides	Asar et al. (2020)
E1andE2	mRNA from ruptured peripheral human atherosclerotic plaques	Target-specific biomarkers	Early and accurate diagnosis of various diseases, including pancreatic cancer and acute myocardial infarction	Diagnostic peptides	Cleutjens et al. (2008)

Recent advances in next-generation sequencing (NGS) have helped address these issues, allowing for the identification of duplicate peptide sequences in early biopanning and accelerating the discovery of specific binders while reducing false-positive hits (Matochko et al., 2014). Next-generation sequencing can also significantly enhance phage display screening by accelerating the discovery of specific binders and limiting the number of false-positive hits ('t Hoen et al., 2012). Deep sequencing can also be used to reduce the risk of identifying false-positive phages before and after each round of biopanning (Matochko et al., 2012; Ito et al., 2023). While there are still challenges to overcome, the potential benefits of phage display screening for functional peptides in pancreatic cancer applications make it an attractive area for continued research and development.

## Summary

Pancreatic cancer is a highly challenging disease with no effective therapies identified to date despite significant progress in understanding its pathobiology. Phage display technology provides a powerful support for early diagnosis, targeted drug delivery, and precision medicine in the asymptomatic stage of pancreatic cancer. Although the application of phage display technology in cancer and oncology has been widely reported (Qu et al., 2017; Li et al., 2020) and some phage display-derived peptides or antibodies for cancer therapy have received FDA approval (Lu et al., 2020; Passariello et al., 2020), the use of phage display-derived peptides in pancreatic cancer has been relatively limited.

This paper is a review of the application of phage display technology in pancreatic cancer research. Phage display is a powerful tool for drug discovery and biomarker imaging, which offers advantages in early detection, targeted drug delivery, and precision medicine for pancreatic cancer. Phage display technology has demonstrated its value as a reliable drug discovery platform, as evidenced by the FDA approval of several anti-cancer drugs derived from phage display, such as avelumab and moxetumomab pasudodox. The article highlights the importance of ongoing investigations using phage display technology in order to make key discoveries for new treatments for pancreatic cancer. The use of phage display technology offers new tools and insights that could ultimately provide hope for pancreatic cancer patients.

## Author contributions

YL: conceptualization and methodology. K-dY: writing-original draft and investigation. J-fY: resources and methodology. Y-nD: validation writing-review and editing. H-yD: writing-review and editing. All authors contributed to the article and approved the submitted version.

## Funding

This study was supported by Scientific Research Project of Jilin Provincial Department of Finance (No.JLSWSRCZX2020-0030; No.JLSWSRCZX2021-074); Research Project of Undergraduate Teaching Reform in Jilin University (No. 2021XZC087); Research Projects of Higher Education in Jilin Province (No. JGJX2021D53).

## Conflict of interest

The authors declare that the research was conducted in the absence of any commercial or financial relationships that could be construed as a potential conflict of interest.

## Publisher’s note

All claims expressed in this article are solely those of the authors and do not necessarily represent those of their affiliated organizations, or those of the publisher, the editors and the reviewers. Any product that may be evaluated in this article, or claim that may be made by its manufacturer, is not guaranteed or endorsed by the publisher.
